# Supplementary figures and images for: Cellf-deception: human microglia clone 3 (HMC3) cells exhibit more astrocyte-like than microglia-like gene expression
Source: Front Bioinform. 2025 Nov 4;5:1681811. doi: 10.3389/fbinf.2025.1681811 (PMC12623408; doi:10.3389/fbinf.2025.1681811)

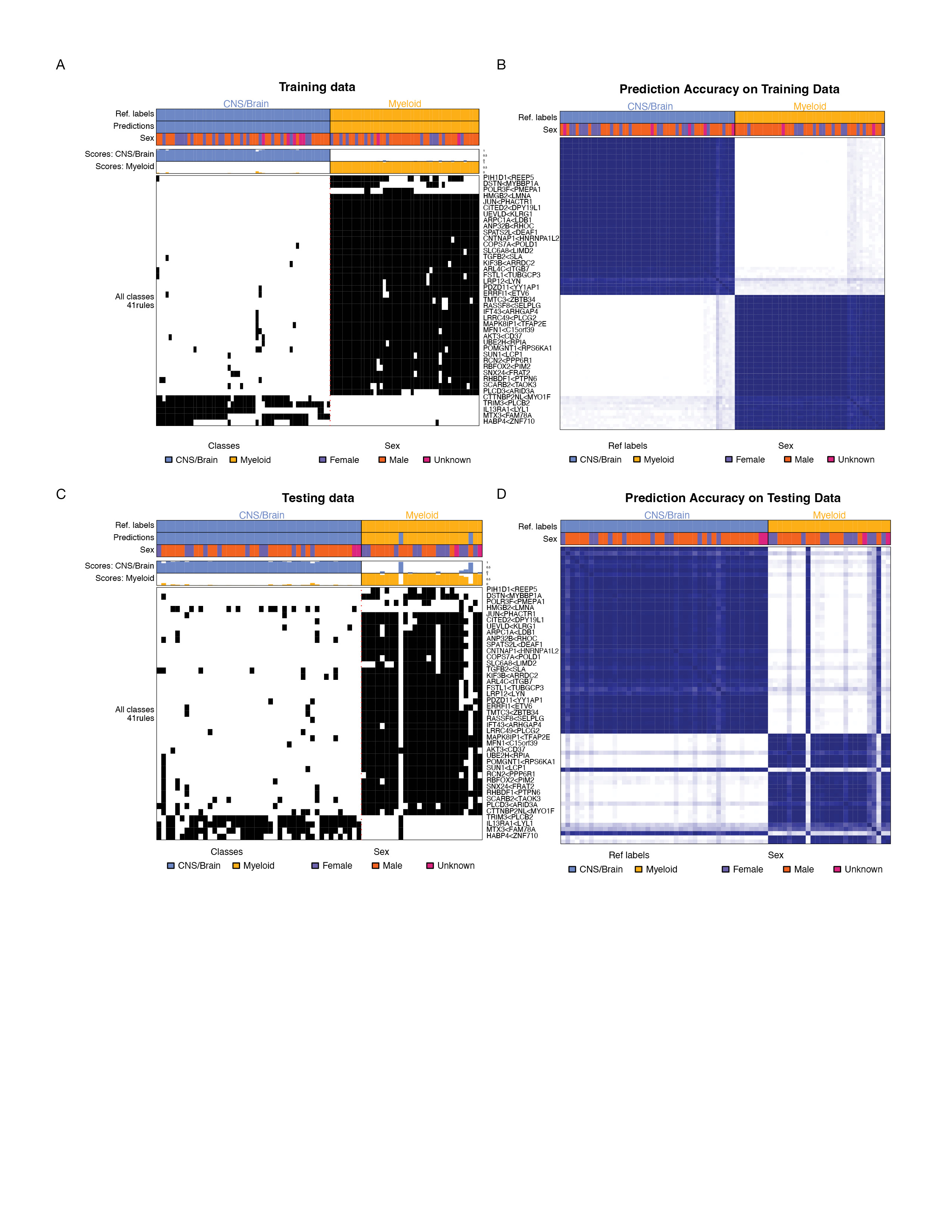

Supplement: Supplementary file 1 [file Image3.jpeg]

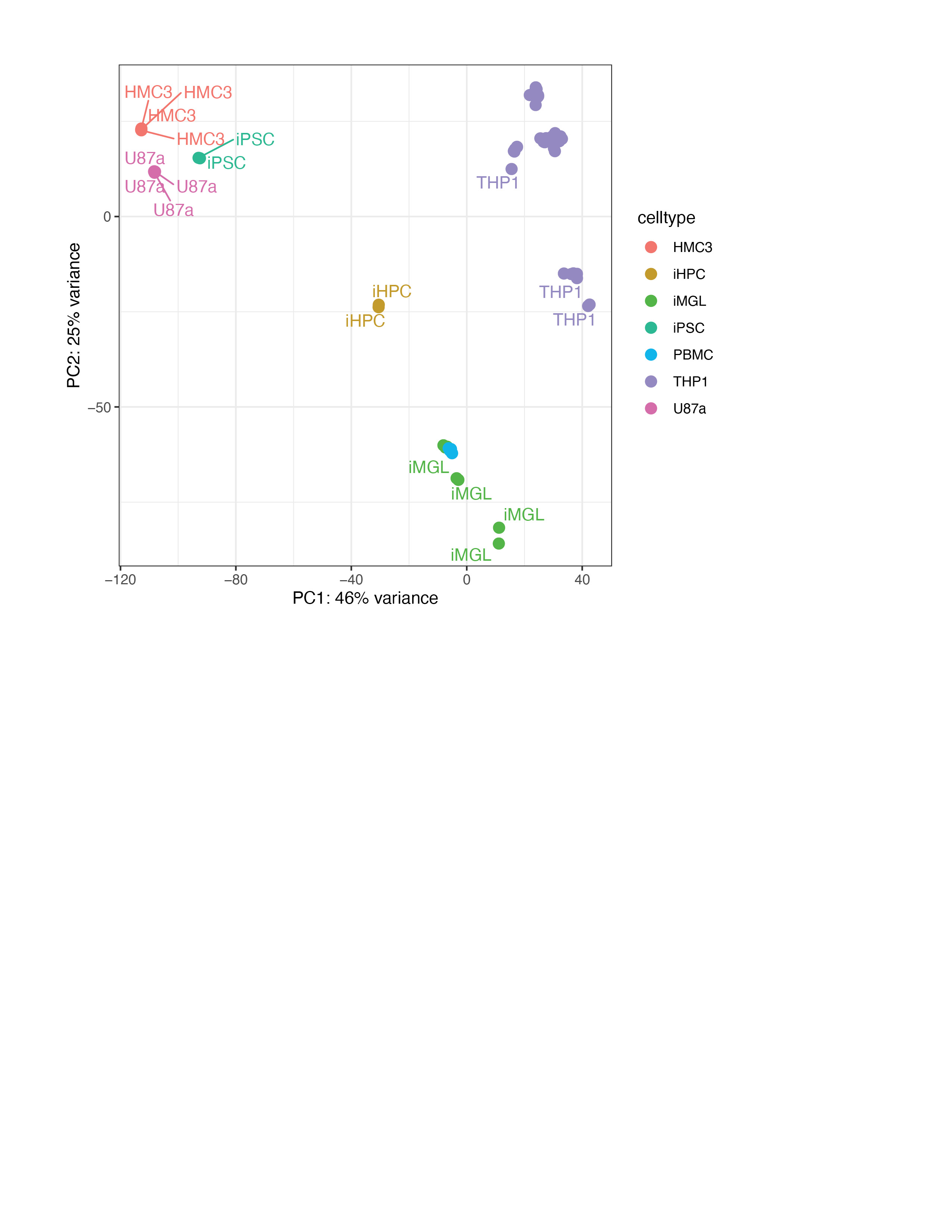

Supplement: Supplementary file 2 [file Image1.jpeg]

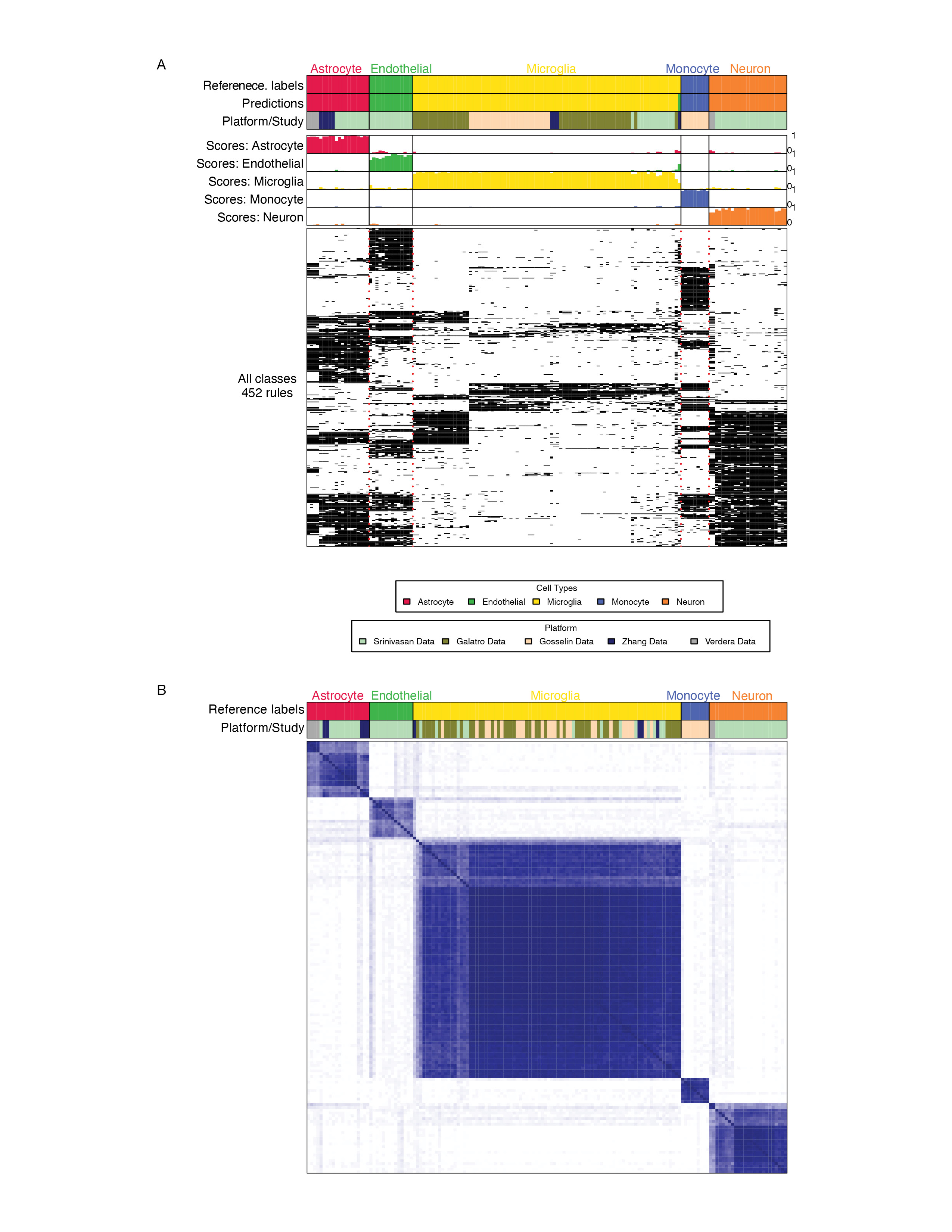

Supplement: Supplementary file 3 [file Image2.jpeg]
